# Integrated Cytokine, Metabolic, and Proliferative Profiling Reveals Divergent Metabolic and Proliferative Responses in Papillary Thyroid Cancer Cells

**DOI:** 10.3390/ijms27146131

**Published:** 2026-07-09

**Authors:** Angelika Buczyńska-Backiel, Julia Redlińska, Julia Zając, Maria Kościuszko, Agnieszka Adamska, Katarzyna Siewko, Anna Popławska-Kita, Adam Jacek Krętowski

**Affiliations:** 1 Clinical Research Centre, Medical University of Bialystok, 15-276 Bialystok, Poland; adamkretowski@wp.pl; 2 Department of Gynecological Endocrinology and Adolescent Gynecology, Medical University of Białystok, 15-276 Białystok, Poland; julia.redlinska@umb.edu.pl; 3 Department of Endocrinology, Diabetology and Internal Medicine, Medical University of Bialystok, 15-276 Bialystok, Poland; julia.zajac@umb.edu.pl (J.Z.); maria.kosciuszko@umb.edu.pl (M.K.); agnieszka.adamska@umb.edu.pl (A.A.); katarzyna.siewko@umb.edu.pl (K.S.); annapoplawskakita@op.pl (A.P.-K.)

**Keywords:** papillary thyroid cancer, angiogenesis, inflammation, cytokines, SGLT2 inhibition, DPP inhibition, metabolism

## Abstract

Papillary thyroid cancer (PTC) exhibits Warburg-type metabolic reprogramming with enhanced glycolysis and dependence on glucose-driven pathways. This study evaluated the effects of antihyperglycemic interventions on cytokine secretion, angiogenic signaling, metabolic activity, and proliferation in thyroid-derived cell models. Two PTC cell lines (MDA-T32 and SCC147) and a normal thyroid line (Nthy-ori) were analyzed for intracellular and extracellular cytokines, secretion efficiency (index), relative metabolic index (RMI), and marker of proliferation (Ki-67) expression following exposure to vandetanib (VDT), sodium–glucose cotransporter 2 (SGLT2), or dipeptidyl peptidase (DPP) inhibitors. Baseline analysis revealed distinct cell line-specific profiles. Compared with Nthy-ori cells, MDA-T32 cells exhibited increased vascular endothelial growth factor (VEGF) concentrations in lysates and conditioned medium (*p* < 0.001, q < 0.001) with enhanced VEGF secretion efficiency (*p* = 0.002, q = 0.008), elevated intracellular fibroblast growth factor (FGF) (*p* < 0.001, q < 0.001) with reduced FGF secretion index (*p* = 0.004, q = 0.01), and lower interleukin 8 (IL-8) concentrations accompanied by increased IL-8 secretion efficiency (*p* = 0.006, q = 0.02). In contrast, SCC147 cells demonstrated reduced VEGF secretion (*p* < 0.001, q < 0.001), decreased intracellular IL-8 (*p* = 0.008, q = 0.02), reduced chemokines of the growth-regulated oncogene GROβ family (GROβ) secretion (*p* = 0.01, q = 0.04), increased IL-8 secretion efficiency (*p* = 0.01, q = 0.03), and decreased GROβ secretion efficiency (*p* = 0.008, q = 0.02). Nthy-ori cells displayed a balanced profile. Among the investigated interventions, VDT produced the most pronounced effects. In MDA-T32 cells, VDT significantly reduced VEGF levels (*p* < 0.001, q < 0.001) and increased IL-8 and GROβ concentrations in conditioned medium (q < 0.05), whereas no significant effects after FDR correction were observed in SCC147 or Nthy-ori cells. SGLT2 and DPP inhibitors produced only nominal effects (*p* < 0.05), which did not remain significant after correction for multiple testing. VDT reduced RMI by approximately 50% in MDA-T32 cells while Ki-67 expression increased, whereas SCC147 cells remained largely unchanged. In Nthy-ori cells, SGLT2 inhibition increased RMI and decreased Ki-67 expression. These findings demonstrate marked heterogeneity among PTC cell lines and suggest that alterations in metabolic activity were not consistently accompanied by proportional changes in proliferative status under the experimental conditions used. VDT predominantly affected angiogenic and inflammatory signaling in MDA-T32 cells, whereas SGLT2 and DPP inhibition exerted limited measurable effects at clinically achievable concentrations.

## 1. Introduction

Papillary thyroid cancer (PTC), although generally associated with a favourable prognosis, includes a subset of tumours that exhibit aggressive behaviour, characterized by angioinvasion, lymph node involvement and resistance to standard therapy [1]. Clinical observations indicate that these aggressive features are closely linked to alterations in the tumour microenvironment, particularly enhanced inflammatory signalling and angiogenic activity [2]. In this context, pro-inflammatory cytokines with angiogenic factors, including vascular endothelial growth factor (VEGF), may play a central role in promoting tumour progression, vascular infiltration and metastatic dissemination [3,4,5,6,7,8,9]. Among inflammatory cytokines, interleukin-8 (IL-8) and chemokines of the growth-regulated oncogene GROβ family (GROβ) act as potent mediators of inflammation, promoting tumour cell migration, neutrophil recruitment and metastatic potential [8,10,11]. At the same time, VEGF serves as a central driver of angiogenesis, directly regulating endothelial cell proliferation, vascular permeability and tumour vascularization [9,12,13]. Additional growth factors, such as platelet-derived growth factor (PDGF) and transforming growth factor alpha (TGFα), contribute to stromal activation and tumour expansion, while fibroblast growth factor (FGF) and epidermal growth factor (EGF) support proliferative and survival signalling [3,10,14]. These mediators define a functional axis linking inflammation and angiogenesis, which is critical for tumour progression and angioinvasion.

Although metabolic reprogramming is widely recognized as a hallmark of cancer and a prerequisite for sustained tumour growth, increasing evidence indicates that cellular proliferation and metabolic activity are not always strictly coupled. Cancer metabolism serves not only to generate ATP but also to provide biosynthetic intermediates required for cell growth and adaptation [15,16,17]. Furthermore, tumour cells can compensate for metabolic stress by activating alternative pathways that maintain proliferation despite reduced metabolic output [18]. Consequently, inhibition of glycolytic activity does not necessarily translate into proportional suppression of tumour growth. Although PTC exhibits enhanced glucose utilization and a glycolytic profile, it remains unclear whether pharmacological modulation of metabolism directly affects proliferative capacity or whether these processes may become partially uncoupled. Addressing this question may improve our understanding of adaptive processes underlying tumour progression and therapeutic resistance.

Therefore, our previous clinical observations demonstrated that increased inflammatory and oxidative stress is associated with more advanced and aggressive PTC profiles, supporting the concept that inflammation and angiogenesis are functionally interconnected processes driving tumour behaviour [2,6,7,19]. These findings may also suggest that modulation of inflammatory–angiogenic signalling may represent a relevant therapeutic strategy in PTC [20]. Notably, inflammatory–angiogenic processes are closely linked to metabolic reprogramming characteristic of the Warburg effect, in which enhanced glycolysis supports both inflammatory signalling and angiogenic activity [17,21,22]. This metabolic–inflammatory–angiogenic axis suggests that targeting glucose metabolism may indirectly modulate tumour-promoting pathways [23]. Accordingly, antihyperglycemic interventions that counteract Warburg-type glycolysis may represent a potential strategy to influence tumour progression by altering the metabolic context underlying inflammatory and angiogenic signalling [20,24,25,26], as glycolytic flux and lactate production directly regulate cytokine secretion and VEGF-driven angiogenic signaling [17,27,28]. Consistent with findings by Coelho et al., demonstrating a glycolytic profile and Warburg-type metabolism in PTC cell lines, characterized by increased glucose consumption, lactate production, and reduced oxidative glucose metabolism, this metabolic dependency provides a rationale for targeting glucose-driven pathways [29].

Pharmacological agents used in the treatment of diabetes-related metabolic disorders, particularly sodium–glucose cotransporter 2 (SGLT2) and dipeptidyl peptidase (DPP) inhibitors, have emerged as potential modulators of these pathways. SGLT2 inhibitors reduce intracellular glucose availability and cellular metabolic flux, which may indirectly suppress pro-angiogenic signalling, including VEGF-dependent pathways [26]. In contrast, DPP inhibitors affect inflammatory signalling and cell–surface proteolytic activity, thereby influencing cytokine networks and immune–tumour interactions [30]. Importantly, both SGLT2 and DPP are dysregulated in PTC tissue, with SGLT2 expression linked to tumour proliferation, invasiveness and adverse clinical outcomes [25,26,31]. Importantly, DPP, also known as CD26, expression was decreased in normal thyroid tissue but significantly upregulated in PTC, where it correlates with tumour aggressiveness, invasiveness and poorer clinical outcomes [30,32,33,34,35]. Moreover, modulation of DPP was suggested as a therapeutic agent in PTC [33]. Crucially, experimental studies have demonstrated that DPP inhibition reduces thyroid cancer cell proliferation, migration and invasive capacity, accompanied by suppression of key signalling pathways involved in tumour progression, including integrin-mediated and AKT-dependent signalling [34]. Likewise, inhibition of SGLT2 has been shown to suppress tumor growth by limiting glucose uptake, inducing oxidative stress, and promoting cell cycle arrest and apoptosis in thyroid cancer models [36,37]. These findings provide a strong mechanistic rationale for targeting SGLT2 and DPP in PTC and suggest that modulation of this molecule may directly impact tumour growth and metastatic potential [34,37]. Despite these observations, it remains unclear whether pharmacological modulation of these pathways can effectively alter the balance between inflammatory and angiogenic signalling in PTC cells [38]. In particular, it is unknown whether such interventions may shift tumour profile toward either a pro-inflammatory or anti-angiogenic state, which could have important implications for tumour progression and therapeutic response [14,39]. To our knowledge, no previous study has simultaneously integrated intracellular and extracellular cytokine profiling, secretion efficiency, metabolic activity, and proliferative status in papillary thyroid cancer models under metabolic interventions. Such an approach may provide additional insight into the relationship between inflammatory signaling, angiogenesis, and the potential dissociation between metabolism and cell growth. Importantly, the DPP inhibitor used in this study was not selective for DPP-4 but also inhibited DPP-8/9, which are intracellular enzymes with distinct biological functions. Unlike DPP-4, DPP-8/9 regulate inflammasome activation, particularly through CARD8 and NLRP1 signaling, leading to caspase-1 activation and pyroptotic cell death. These pathways are strongly associated with pro-inflammatory responses and may therefore influence inflammatory signaling independently of canonical DPP-4–related mechanisms. Consequently, simultaneous inhibition of DPP-8/9 may introduce competing or compensatory signaling effects, potentially masking the expected effects of selective DPP-4 inhibition. Notably, the role of DPP-8/9 in thyroid cancer remains largely unexplored [40,41,42].

Therefore, the aim of this study was to evaluate the effects of SGLT2 and DPP inhibition on cytokine and growth factor profiles in PTC cell lines and normal thyroid cells. By analysing intracellular (lysates) and secreted (conditioned medium) levels, as well as secretion efficiency (index), we assessed whether these interventions differentially modulate inflammatory and angiogenic pathways, thereby identifying cell line-specific profiles relevant to tumour aggressiveness and angioinvasion. However, despite the recognized dependence of PTC on glucose metabolism, it remains unclear whether alterations in metabolic activity necessarily translate into proportional changes in proliferative capacity. Therefore, an important question of this study was whether metabolism and cell growth remain tightly coupled in PTC cells or whether these processes may become partially uncoupled under pharmacological intervention.

## 2. Results

**Figure 1:**
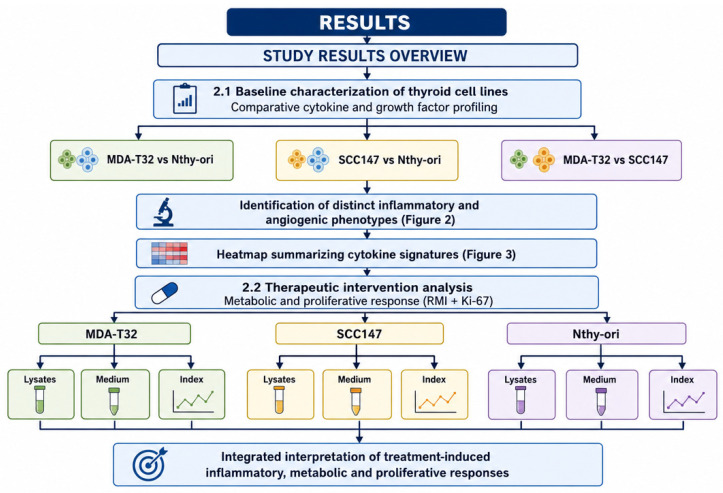
Graphical roadmap of the Results section illustrating the sequential organization of the analyses. The Results progress from baseline characterization of thyroid cell lines, through identification of inflammatory and angiogenic phenotypes, to treatment-specific analyses and integrated metabolic–proliferative interpretation. This overview is intended to facilitate navigation through the complex experimental workflow before presentation of the detailed quantitative results.

Given the complexity of the experimental design, the Results are presented in a stepwise manner to facilitate interpretation. The section first establishes the baseline inflammatory and angiogenic phenotypes of the analyzed thyroid cell lines by comparing intracellular cytokine expression, secreted protein concentrations, and secretion efficiency. This biological characterization provides the foundation for the subsequent analyses. The effects of the investigated therapeutic interventions are then presented separately for each cell line using the same analytical sequence (intracellular proteins, secreted proteins, secretion indices, and integrated metabolic–proliferative assessment), allowing direct comparison between experimental models. This organization was intentionally chosen to provide a logical progression from descriptive characterization of the models to mechanistic interpretation of treatment-induced responses while preserving the complete quantitative dataset (Figure 1). Throughout the analysis, nominal significance (*p* < 0.05) was distinguished from statistical significance after FDR correction (q < 0.05).

### 2.1. Inflammatory Models Evaluation

Comparative analysis of cytokine and growth factor profiles revealed distinct differences between MDA-T32, SCC147, and control Nthy-ori cells at both intracellular and secreted levels.

a. MDA-T32 vs. Nthy-ori

VEGF levels were significantly increased in MDA-T32 cells compared to Nthy-ori in both lysates and conditioned medium (*p* < 0.001, q < 0.001 for both). FGF levels were significantly higher in lysates (*p* < 0.001, q < 0.001), with no significant differences observed in conditioned medium. In contrast, IL-8 levels were significantly decreased in MDA-T32 cells in both lysates (*p* = 0.004) and conditioned medium (*p* = 0.003) (Supplementary Table S1).

Index analysis showed increased VEGF secretion efficiency in MDA-T32 cells (*p* = 0.002, q = 0.008), as well as increased IL-8 index (*p* = 0.006, q = 0.02). In contrast, FGF index was significantly decreased (*p* = 0.004, q = 0.01) (Supplementary Table S2).

b. SCC147 vs. Nthy-ori

VEGF levels in conditioned medium were significantly decreased in SCC147 cells compared to Nthy-ori (*p* < 0.001), while no significant differences were observed after FDR correction in lysates. IL-8 levels were significantly reduced in lysates (*p* = 0.008, q = 0.02), and GROβ levels were decreased in conditioned medium (*p* = 0.01, q = 0.04; Supplementary Table S3).

Index analysis showed a significant decrease in VEGF secretion efficiency (*p* < 0.001, q < 0.001; Supplementary Table S3). In contrast, IL-8 index was increased (*p* = 0.01, q = 0.03), while GROβ index was decreased (*p* = 0.008, q = 0.02) (Supplementary Table S4).

c. MDA-T32 vs. SCC147

VEGF levels were significantly higher in MDA-T32 cells compared to SCC147 in both lysates and conditioned medium (*p* < 0.001 for both). In contrast, IL-8 levels were significantly higher in SCC147 cells in both lysates and conditioned medium (*p* < 0.001).

GROβ and TGFα levels in conditioned medium were significantly increased in MDA-T32 cells compared to SCC147 (*p* = 0.01, q = 0.03 for both), while FGF levels were higher in MDA-T32 lysates (*p* < 0.001) (Supplementary Table S5).

Index analysis showed increased VEGF secretion efficiency in MDA-T32 cells (*p* < 0.001, q < 0.001), as well as increased GROβ (*p* = 0.008, q = 0.02), IL-8 (*p* = 0.01, q = 0.03), and TGFα (*p* = 0.01, q = 0.03) indices. In contrast, FGF index was decreased in MDA-T32 cells (*p* = 0.004, q = 0.01) (Supplementary Table S6).

d. Summary and characterization of cell line profiles

**Figure 2:**
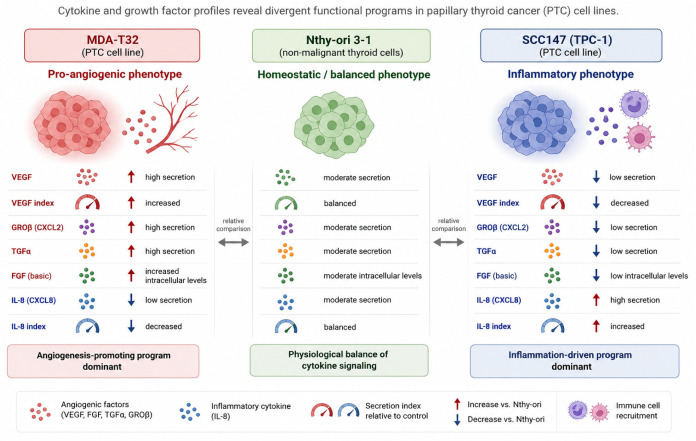
Distinct inflammatory and angiogenic profiles of the analyzed thyroid cell lines.

The comparative analysis of cytokine and growth factor profiles revealed that the three analyzed cell lines represent distinct biological profiles, differing primarily in angiogenic potential and inflammatory signalling (Figure 2).

i. MDA-T32—pro-angiogenic and highly secretory profile

MDA-T32 cells are characterized by a predominantly pro-angiogenic profile, driven by significantly increased VEGF production and secretion. This was consistently observed across intracellular, extracellular, and index-based analyses, indicating both enhanced synthesis and efficient release of VEGF. In addition, MDA-T32 cells exhibited elevated secretion of GROβ and TGFα, suggesting active communication with the tumor microenvironment. Despite lower absolute IL-8 levels compared to control cells, MDA-T32 demonstrated increased IL-8 secretion efficiency, indicating a regulated and effective cytokine release. Conversely, FGF was significantly elevated intracellularly but not secreted, suggesting retention within the cells. Collectively, these findings describe a cytokine secretion profile consistent with enhanced angiogenic signaling under the experimental conditions studied.

ii. SCC147—inflammatory, non-angiogenic profile

SCC147 cells displayed a markedly different profile. The most prominent feature was the near-complete loss of VEGF secretion, despite relatively preserved intracellular levels, indicating a defect or suppression in secretion pathways. At the same time, SCC147 cells showed higher IL-8 levels compared to MDA-T32, along with increased IL-8 secretion efficiency, pointing to a more pronounced inflammatory profile. Additionally, reduced GROβ secretion suggests altered signaling toward the microenvironment. These features define SCC147 as a predominantly non-angiogenic but rather inflammatory tumor profile, potentially relying on alternative mechanisms of tumor progression independent of VEGF-driven angiogenesis.

iii. Nthy-ori—balanced, homeostatic profile

**Figure 3:**
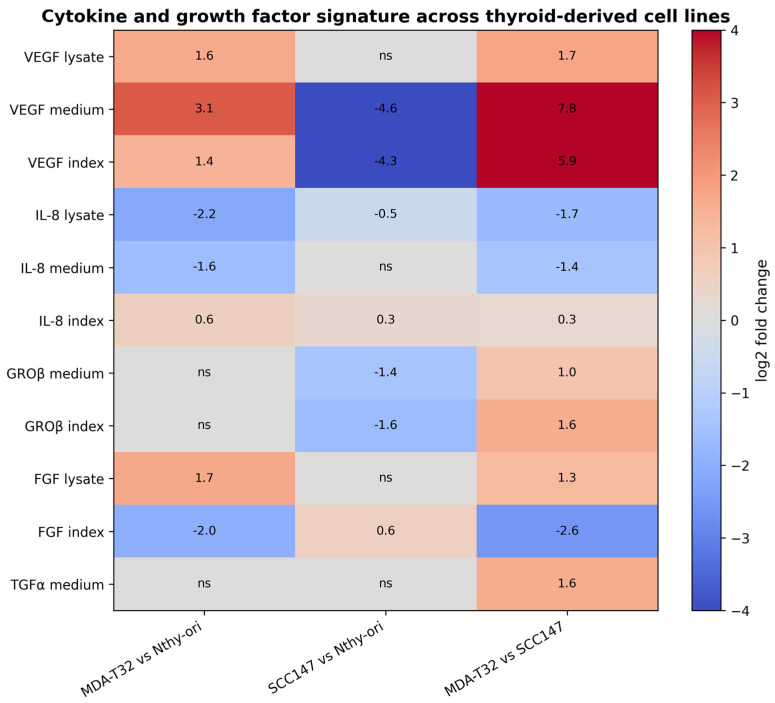
Heatmap summarizing the cytokine and growth factor signatures across thyroid cell lines. “ns” indicates non-significant differences.

Control Nthy-ori cells exhibited an intermediate and balanced profile, with moderate VEGF secretion and relatively high IL-8 production. Importantly, secretion efficiency remained stable across most markers, reflecting a homeostatic, non-transformed cellular state (Figure 3).

### 2.2. Therapeutic Intervention Analysis

a. Integrated metabolic and proliferative assessment (Ki67)

**Figure 4:**
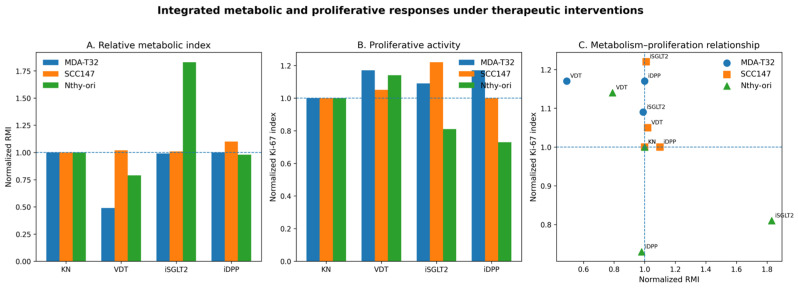
Integrated metabolic and proliferative responses under therapeutic interventions.

The RMI showed distinct, cell line-dependent changes. In MDA-T32 cells, VDT treatment resulted in a marked decrease in RMI (50% reduction), indicating reduced lactate production relative to intracellular glucose levels. No substantial changes were observed following SGLT2 or DPP inhibitors. In SCC147 cells, RMI values remained stable across all conditions, indicating a lack of metabolic response to treatment. In Nthy-ori cells, VDT treatment was associated with a moderate decrease in RMI, whereas inhibitor of SGLT2 resulted in a marked increase, indicating enhanced lactate production relative to glucose availability (Table 1, Figure 4). RMI reflects relative metabolic state rather than direct glycolytic flux and should be interpreted as an indirect surrogate. The purpose of introducing the RMI was not to validate a novel metabolic biomarker but to provide a standardized comparative index allowing consistent evaluation of relative metabolic changes across experimental conditions.

b. Effect of therapeutic interventions in MDA-T32

Therapeutic interventions affected cytokine and growth factor levels in MDA-T32 cells at both intracellular and secreted levels.

i. Lysates (intracellular levels)

VDT treatment significantly reduced VEGF levels (*p* < 0.001, q < 0.001; Table 2). GROβ and IL-8 levels were increased (*p* = 0.02 and *p* = 0.03, respectively); however, these changes were not significant after FDR correction. FGF levels were decreased (*p* = 0.02), without significance after multiple testing correction. No statistically significant changes after FDR correction were observed following SGLT2 or DPP inhibitors (Table 2).

ii. Conditioned medium (secreted levels)

VDT treatment significantly reduced VEGF levels in conditioned medium (*p* < 0.001, q < 0.001; Table 3). IL-8 and GROβ levels were increased (*p* = 0.01 and *p* = 0.008, respectively), and both remained significant after FDR correction (q < 0.05). DPP inhibition decreased IL-8 levels (*p* = 0.03); however, this change was not significant after FDR correction. No statistically significant changes after FDR correction were observed following the SGLT2 inhibitor. Only decreased VEGF levels in lysates and conditioned medium following VDT, as well as increased IL-8 and GROβ levels in conditioned medium following VDT, remained significant after FDR correction. All other observed differences were not significant after multiple testing correction (Table 3 and Table 4).

c. Effect of therapeutic interventions in SCC147

Therapeutic interventions had limited effects on cytokine and growth factor levels in SCC147 cells in both intracellular and secreted fractions.

i. Lysates (intracellular levels)

VDT treatment resulted in changes in intracellular protein levels; however, none of the observed differences were statistically significant after FDR correction (Table 5). Nominal decreases (*p* < 0.05) were observed for IL-8, GROβ, VEGF, PDGF, and TGFα, but these did not remain significant after multiple testing correction. No statistically significant changes after FDR correction were observed following ISGLT2 or IDPP inhibitors (Table 5).

ii. Conditioned medium (secreted levels)

In conditioned medium, VDT treatment resulted in increased IL-8 and GROβ levels (*p* < 0.05), with additional nominal increases observed for VEGF and FGF (Table 6); however, none of these changes remained significant after FDR correction, despite several markers showing nominal significance (*p* < 0.05). DPP inhibitor stimulation was associated with a decrease in IL-8 levels (*p* < 0.05), while no significant changes were observed following SGLT2 inhibitors stimulation. No statistically significant differences were detected for any marker after multiple testing correction (Table 6). Index analysis showed no statistically significant changes after FDR correction. However, borderline differences were observed for selected markers (e.g., FGF), which reached nominal significance (*p* < 0.05) but did not remain significant after multiple testing correction (Table 7).

d. Effect of therapeutic interventions in Nthy-ori (control cells)

Therapeutic interventions resulted in changes in cytokine and growth factor levels in Nthy-ori cells; however, none of the observed differences were statistically significant after FDR correction.

i. Lysates (intracellular levels)

VDT treatment was associated with nominal decreases in IL-8, GROβ, VEGF, and FGF levels (*p* < 0.05), with additional trends observed for PDGF and TGFα (Table 8). However, none of these changes remained significant after FDR correction (Table 8). No statistically significant changes after FDR correction were observed following SGLT2 or DPP inhibitors.

ii. Conditioned medium (secreted levels)

In conditioned medium, VDT treatment resulted in nominal decreases in IL-8, GROβ, and VEGF levels (*p* < 0.05; Table 9); however, these changes did not remain significant after FDR correction. No statistically significant differences were observed following SGLT2 or DPP inhibitors. Index analysis showed no statistically significant differences after FDR correction. However, several markers (including IL-8, VEGF, and FGF) showed nominal significance (*p* < 0.05) (Table 10).

e. Integrated analysis of metabolic, proliferative, and cytokine profiles

**Figure 5:**
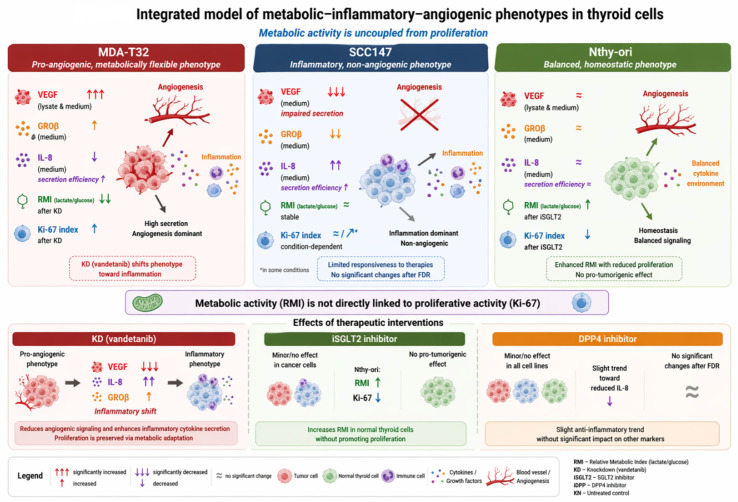
Integrated model of metabolic–inflammatory–angiogenic profiles in thyroid-derived cell lines and their response to therapeutic interventions. The schematic summarizes functional differences between papillary thyroid cancer cell lines (MDA-T32, SCC147) and normal thyroid cells (Nthy-ori) in terms of cytokine secretion, angiogenic signaling, metabolic activity, and proliferation. MDA-T32 cells exhibit a predominantly pro-angiogenic profile characterized by elevated VEGF levels and high secretion activity, with a shift toward inflammatory signaling upon vandetanib (VDT; vandetanib). SCC147 cells display an inflammatory, non-angiogenic profile with markedly reduced VEGF secretion and increased IL-8 secretion efficiency, accompanied by limited responsiveness to therapeutic interventions. Nthy-ori cells maintain a balanced, homeostatic profile with stable cytokine expression and regulated secretion. Integration of metabolic and proliferative data indicates a dissociation between relative metabolic activity and proliferation. In MDA-T32 cells, VDT reduces the relative metabolic index (RMI) while increasing Ki-67 expression, suggesting preserved proliferative capacity despite reduced metabolic output. In contrast, ISGLT2 inhibition in Nthy-ori cells increases RMI but is associated with reduced proliferation, indicating that enhanced metabolic activity does not directly promote cell growth in non-malignant cells. Therapeutic interventions exert cell line-specific effects. VDT shifts MDA-T32 cells from an angiogenic toward a predominantly inflammatory profile, whereas ISGLT2 and IDPP inhibition show minimal or no significant effects across cell lines after correction for multiple comparisons. Overall, the model highlights metabolic plasticity and the dynamic interplay between angiogenesis, inflammation, and proliferation in thyroid cancer.

To facilitate interpretation of the complex interactions between cytokine secretion, metabolic activity, and proliferation, an integrated model summarizing the observed profiles across all cell lines and treatment conditions is presented (Figure 5). This model highlights distinct functional profiles, with MDA-T32 cells exhibiting a predominantly pro-angiogenic profile, SCC147 cells displaying an inflammatory, non-angiogenic profile, and Nthy-ori cells maintaining a balanced, homeostatic state. Importantly, integration of metabolic and proliferative parameters revealed a dissociation between relative metabolic activity, assessed by the RMI, and proliferation, assessed by the Ki-67 index. In MDA-T32 cells, vandetanib (VDT) resulted in a reduction of RMI accompanied by an increase in Ki-67, indicating preserved proliferative capacity despite decreased metabolic output. In contrast, SCC147 cells demonstrated a relatively stable metabolic profile with condition-dependent variation in proliferation, while Nthy-ori cells showed increased RMI but reduced proliferation following SGLT2 inhibition. These findings support the concept of metabolic plasticity and suggest that metabolic activity is not directly coupled to proliferative capacity in thyroid-derived cells under the studied conditions. The integrated model further illustrates that therapeutic interventions exert cell line-specific effects, with VDT inducing a shift from angiogenic to inflammatory signaling in MDA-T32 cells, whereas SGLT2 and DPP inhibition resulted in minimal or no significant changes after correction for multiple comparisons. The lack of significant effects may suggest that metabolic targeting alone would be insufficient to modulate cytokine-driven signaling in PTC. The proposed classifications are intended as descriptive tools facilitating comparison between cell lines and should not be interpreted as evidence of discrete or biologically validated phenotypic entities.

## 3. Discussion

This study represents, to our knowledge, one of the first integrated cytokine, metabolic, and proliferative profiles in PTC, revealing that reductions in metabolic activity were not consistently accompanied by proportional changes in proliferative status under the experimental conditions. In MDA-T32 cells, VDT treatment resulted in a marked reduction in the RMI, indicating decreased glycolytic activity, while simultaneously increasing the Ki-67 proliferation index. This suggests that proliferative activity in these cells may be maintained independently of glycolytic flux, potentially through alternative metabolic pathways such as oxidative phosphorylation or glutamine metabolism [43]. In contrast, SCC147 cells exhibited stable RMI and Ki-67 values across all treatment conditions, indicating metabolic and proliferative resistance to therapeutic modulation. This finding is consistent with the observed resistance of this cell line to cytokine and angiogenic changes, further supporting the concept of a distinct, therapy-resistant profile. Interestingly, in Nthy-ori cells, increased RMI following SGLT2 inhibition was accompanied by a reduction in proliferation, suggesting that enhanced glycolytic output does not necessarily translate into increased proliferative activity in non-malignant cells. Therefore, these findings indicate that metabolic reprogramming in thyroid cancer is not directly coupled to proliferation and may represent an independent axis of tumor adaptation. This metabolic plasticity may potentially contribute to adaptive responses and reduced treatment sensitivity and highlights the need for integrated targeting of both metabolic and signaling pathways [44]. The observed dissociation between metabolic activity and proliferation has important clinical implications. In MDA-T32 cells, reduced RMI, reflecting lower lactate production relative to intracellular glucose levels following VDT treatment, did not translate into decreased proliferation, suggesting the presence of metabolic flexibility. This phenomenon, often described in aggressive cancers, enables tumor cells to switch between metabolic pathways such as glycolysis and oxidative phosphorylation to sustain growth despite therapeutic pressure [15,22,45]. In contrast, SCC147 cells exhibited both metabolic and proliferative resistance to treatment, supporting the concept of intrinsically resistant tumor profiles. Such resistance has been associated with reduced dependence on glycolysis and increased reliance on alternative signaling pathways, including inflammatory networks [15]. Interestingly, in Nthy-ori cells, increased glycolytic activity following SGLT2 inhibition was accompanied by reduced proliferation, suggesting that metabolic activation does not necessarily promote tumor-like growth in non-malignant cells. This observation aligns with clinical data indicating that SGLT2 inhibitors do not increase cancer risk and may even exert protective or anti-proliferative effects [46,47]. Previous studies have shown that SGLT2 inhibition can limit glucose uptake in cancer cells and reduce tumor growth through metabolic stress [47], whereas DPP inhibition has been linked to modulation of immune and inflammatory pathways rather than direct effects on tumor metabolism [48,49]. However, our results indicate that these mechanisms may not directly translate into short-term changes in cytokine secretion or proliferation in vitro. Collectively, these findings suggest that targeting glucose metabolism alone may be insufficient to suppress tumor growth, particularly in metabolically adaptable cancers. Combined therapeutic strategies integrating metabolic inhibition with modulation of inflammatory and angiogenic pathways may therefore represent a more effective approach in adjuvant PTC treatment research. These observations are in line with the findings reported by Coelho et al., who demonstrated that PTC cell lines exhibit a pronounced glycolytic profile characterized by increased glucose consumption, elevated lactate production, reduced oxidative glucose metabolism, and increased ROS, consistent with the Warburg effect. Importantly, the study also highlighted metabolic heterogeneity between cell lines, including differential engagement of glycolysis and the pentose phosphate pathway [29]. This is consistent with our findings, where distinct metabolic and functional profiles were observed between MDA-T32 and SCC147 cells. Notably, despite evidence of glycolytic activity, metabolic modulation did not uniformly translate into changes in cytokine secretion or proliferative capacity, supporting the concept of metabolic plasticity and suggesting that glycolysis-driven signaling may be differentially coupled to inflammatory and angiogenic pathways depending on cellular context.

The present study identified distinct functional profiles that may reflect underlying tumor progression and angioinvasion. Baseline comparisons revealed clear heterogeneity between the analyzed cell lines. MDA-T32 cells were characterized by significantly increased VEGF levels and enhanced secretion efficiency, indicating a strongly angiogenic and highly secretory profile. In contrast, SCC147 cells exhibited markedly reduced VEGF secretion despite preserved intracellular levels, suggesting dysregulation at the level of secretion rather than synthesis. At the same time, SCC147 cells demonstrated increased IL-8 secretion efficiency and reduced GROβ release, consistent with a predominantly inflammatory, non-angiogenic profile. Nthy-ori cells displayed a balanced profile with stable secretion patterns. These observations are consistent with previous findings indicating that inflammatory and oxidative stress pathways are closely associated with angioinvasion and aggressive behavior in PTC [2,9,14,39]. Our earlier clinical studies demonstrated that increased oxidative stress markers and endothelial dysfunction correlate with angioinvasive features and metastatic potential in PTC [2,6,7,50]. Similarly, cytokines such as IL-8 and GROβ family chemokines have been shown to promote tumor progression, migration, and microenvironment remodeling, while VEGF remains a central regulator of angiogenesis [51,52,53]. The observed inverse relationship between VEGF-driven angiogenesis and IL-8–associated inflammatory signaling suggests the presence of a functional balance between these pathways [12,54]. MDA-T32 cells exhibited dominant VEGF signaling, whereas SCC147 cells appeared to rely more on inflammatory mediators. This supports the concept that tumor cells may adopt alternative strategies for progression, either through classical angiogenesis or through inflammation-driven pathways that may partially compensate for impaired vascular signaling. Such plasticity has been described in multiple tumor types and is increasingly recognized as a factor contributing to therapeutic resistance [44,55,56]. Among the tested interventions, only VDT treatment (vandetanib) produced statistically significant effects after FDR correction, particularly in MDA-T32 cells. VDT markedly reduced VEGF levels in both intracellular and extracellular compartments, confirming effective inhibition of angiogenic signaling pathways. This is consistent with the known mechanism of VDT, which targets VEGFR, EGFR, and RET signaling and suppresses tumor growth and vascularization. This observation is consistent with clinical evidence supporting the efficacy of VDT in advanced thyroid cancer. In a randomized, double-blind phase 2 trial conducted by Martin J. Schlumberger et al., VDT significantly prolonged progression-free survival in patients with locally advanced or metastatic differentiated thyroid carcinoma compared to placebo (median PFS 11.1 vs. 5.9 months; *p* = 0.008). The therapeutic benefit was attributed to inhibition of VEGFR, EGFR, and RET signaling pathways, confirming the central role of angiogenic signaling in disease progression [57]. Our findings at the cellular level are in line with these clinical observations, demonstrating that VDT effectively suppresses VEGF expression and secretion [58]. However, the concomitant increase in IL-8 and GROβ secretion observed in our study suggests that inhibition of angiogenesis may be accompanied by activation of compensatory inflammatory pathways [9,14,52,53]. This mechanism could potentially contribute to adaptive resistance and may partially explain the limited durability of response observed in some patients treated with tyrosine kinase inhibitors [39,57,58]. Importantly, the present study was designed to identify phenotypic patterns rather than establish causal molecular mechanisms. Therefore, the proposed biological interpretations should be considered hypothesis-generating and require validation using dedicated functional approaches, including pathway-specific inhibition, genetic manipulation, or rescue experiments.

In contrast, neither SGLT2 nor DPP inhibition resulted in statistically significant changes after FDR correction in any of the analyzed cell lines. Although nominal changes were observed, particularly in inflammatory markers, these findings should be interpreted in the context of experimental exposure and inhibitor specificity. Previous studies demonstrating anticancer effects of SGLT2 inhibition in thyroid cancer models reported suppression of tumor growth through reduced glucose uptake, induction of oxidative stress, and cell cycle arrest; however, these effects were typically observed at micromolar concentrations that exceed those used in the present study [37,47,59]. In contrast, the present study employed dapagliflozin at 0.1 µM, a concentration within the clinically achievable range, which may partly explain the absence of significant effects after multiple testing correction. Importantly, the DPP inhibitor used in the present study was a non-selective compound targeting not only DPP-4 but also DPP-8/9. While most studies in thyroid cancer have focused on selective DPP-4 inhibition, which has been associated with reduced proliferation, migration, and invasive capacity [30,33,34], DPP-8/9 are involved in the regulation of inflammasome activation and inflammatory cell death pathways [60]. Therefore, concurrent inhibition of DPP-8/9 may induce pro-inflammatory signaling and modify or counteract the effects attributed to DPP-4 inhibition [61]. This lack of selectivity may contribute to the absence of statistically significant changes after FDR correction, as overlapping or opposing signaling mechanisms could mask measurable effects on cytokine secretion and angiogenic signalling [40]. Overall, these findings suggest that the lack of significant effects does not necessarily reflect the absence of biological activity, but rather highlights the importance of drug concentration, exposure conditions, and target specificity in interpreting the effects of metabolic interventions in PTC models. A key observation of this study is the strong dependence of treatment response on cellular context. MDA-T32 cells, characterized by a predominantly pro-angiogenic profile, showed clear sensitivity to kinase inhibition, whereas SCC147 cells, representing a predominantly inflammatory profile, exhibited resistance to all tested interventions. Nthy-ori control cells remained largely unaffected, supporting the specificity of observed effects in malignant cells. These differences highlight the presence of distinct biological vulnerabilities among thyroid cancer subtypes and suggest that therapeutic strategies should be tailored to the dominant signaling axis within a given tumor.

Several limitations should be acknowledged. The study was conducted in in vitro models, which do not fully recapitulate the complexity of the tumor microenvironment. The proposed cytokine-based cell profiles were inferred from biochemical markers only and were not validated using functional assays such as migration, invasion, or angiogenesis models. Additionally, the use of a non-selective DPP inhibitor represents an important limitation, as simultaneous inhibition of DPP-8/9 may have influenced inflammatory signaling independently of DPP-4–related mechanisms. The sample size was limited, as the analyses were based on three independent biological experiments, each performed in technical replicates. Although FDR correction was applied, some biologically relevant effects may not have reached statistical significance. Due to the small number of biological replicates and non-normal data distribution, non-parametric statistical methods (Mann–Whitney U test) were applied, which, while appropriate for the data structure, may have limited statistical power to detect subtle differences. Additionally, functional outcomes such as cell migration, invasion, or angiogenic capacity were not directly assessed, and no functional validation experiments were performed to confirm the biological impact of the observed cytokine and metabolic changes. Importantly, metabolic activity was evaluated using a RMI, which does not directly reflect glycolytic flux, glucose uptake, or mitochondrial respiration, and should therefore be interpreted as an indirect measure of metabolic state. Furthermore, total protein content was assessed using spectrophotometric measurement (NanoDrop), rather than colorimetric assays such as BCA, which may affect the precision of normalization. In addition, drug concentrations were selected based on literature data and preliminary viability assays to reflect clinically achievable exposure, and no dose–response analysis or IC_50_ determination was performed. Therefore, potential concentration-dependent effects may not have been fully captured. Finally, the short-term exposure to pharmacological agents may not reflect long-term adaptive responses, and the use of established cell lines may not fully represent interpatient heterogeneity observed in clinical PTC.

In conclusion, the present study demonstrates that PTC cells exhibit distinct inflammatory and angiogenic profiles that are differentially modulated by therapeutic interventions. VDT treatment effectively suppresses VEGF-dependent signaling in angiogenic cells but is associated with activation of inflammatory pathways, suggesting potential compensatory mechanisms. In contrast, SGLT2 and DPP inhibition showed limited effects on cytokine profiles under the tested conditions. We believe that the absence of statistically robust effects after FDR correction constitutes an important biological observation in itself and should not be interpreted as a weakness of data reporting but rather as an objective outcome of the experimental study. Thus, these findings support the concept that tumor behavior in PTC is driven by a dynamic balance between angiogenesis and inflammation, which may play a critical role in angioinvasion and therapeutic response. Moreover, these findings suggest that inhibition of angiogenesis alone may be insufficient, and combined targeting of inflammatory signaling pathways could represent a more effective therapeutic strategy. Nevertheless, these findings should be interpreted as exploratory due to the limited sample size.

## 4. Material and Methods

### 4.1. Cell Lines and Culture Conditions

Three human thyroid-derived cell lines were included: two PTC models, SCC147 (TPC-1, Merck, Darmstadt, Germany) and MDA-T32 (CRL-3351, ATCC, Manassas, VA, USA), as well as the non-malignant thyroid follicular cell line NTHY-ORI (Nthy-ori 3-1, Merck, Darmstadt, Germany). Cell cultures were regularly inspected for morphology and viability and subcultured before reaching full confluence.

Cells were maintained in RPMI-1640 medium supplemented with 10% fetal bovine serum (FBS) and 1% penicillin/streptomycin under standard culture conditions (37 °C, 5% CO_2_, humidified atmosphere). For experimental procedures, the serum concentration was reduced to 2.5% FBS to minimize the influence of exogenous growth factors that could interfere with metabolic and redox measurements.

#### 4.1.1. Reagents and Inhibitors

The study employed three pharmacological modulators: an SGLT2 inhibitor, DPP inhibitor and vandetanib (VDT), used as a reference tyrosine kinase inhibitor. Stock solutions were prepared in sterile phosphate-buffered saline (PBS) in accordance with the manufacturer’s guidelines and diluted in culture medium immediately before application.

Final concentrations were selected based on published pharmacokinetic data and preliminary MTT viability assays to approximate clinically achievable plasma concentrations while avoiding overt cytotoxicity:

- SGLT2 inhibitor: 1 × 10^−7^ M (dapagliflozin, Sigma-Aldrich, Merck, Darmstadt, Germany)
- DPP inhibitor: 1 × 10^−6^ M (IDPP inhibitor, Sigma-Aldrich, Merck, Darmstadt, Germany)
- VDT: 1 × 10^−6^ M (vandetanib, Sigma-Aldrich, Merck, Darmstadt, Germany)

The inhibitor of DPP used (Inhibitor-DPP-IV-IN-2) is a non-selective experimental compound that targets both DPP-4 and DPP-8/9. VDT was included as a reference compound due to its inhibitory activity against RET, VEGFR2, and EGFR signaling pathways. Through modulation of PI3K/AKT and MAPK signaling, VDT reduces cellular metabolic activity and glycolytic flux, consistent with previously reported effects on metabolic indices. In addition, tyrosine kinase inhibition may impact mitochondrial function and reactive oxygen species (ROS) production.

Control conditions (KN) consisted of cells treated with vehicle only (PBS) at volumes equivalent to those used for drug administration.

#### 4.1.2. Cell Viability Assay (MTT)

To identify non-cytotoxic concentrations suitable for metabolic analyses, preliminary viability testing was performed using the MTT assay (V13154, Invitrogen, Thermofisher, Waltham, MA, USA). This approach was designed to evaluate metabolic modulation rather than cytotoxicity or antiproliferative effects.

Cells were seeded in 96-well plates at a density of 3000 cells per well and allowed to attach for 24 h. Subsequently, cells were exposed to a range of concentrations of SGLT2 inhibitor, DPP inhibitor, and VDT for 24 and 48 h. After incubation, MTT reagent was added and processed according to the manufacturer’s protocol. Formazan crystals were dissolved and absorbance was measured using a microplate reader.

Concentrations were selected based on preliminary viability assays and previously published pharmacokinetic data to reflect clinically achievable exposure levels (SGLT2 inhibitor 10^−7^ M, DPP inhibitor 10^−6^ M, VDT 10^−6^ M). The MTT assay was used solely to exclude toxic conditions and not to determine dose–response relationships or IC_50_ values. Selected concentrations correspond to clinically relevant plasma levels previously reported in literature [59,62,63,64,65,66,67].

#### 4.1.3. Experimental Design and Treatments

For the main experimental setup, cells were seeded in 6-well plates at a density of 1 × 10^6^ cells per well in complete RPMI-1640 medium and allowed to adhere overnight. The following treatment conditions were applied for 48 h:

- KN: control (PBS)
- SGLT2 inhibitor: 1 × 10^−7^ M
- DPP inhibitor: 1 × 10^−6^ M
- VDT: 1 × 10^−6^ M

Following incubation, conditioned media were collected, centrifuged to remove debris, and stored for further analysis. Cells were washed with PBS and processed to obtain lysates for biochemical assays and protein quantification.

All experiments were performed in three independent biological replicates. Within each independent experiment, measurements were obtained in technical triplicates, and the mean of these technical replicates was used as one biological replicate for further analysis. Final data are presented as median values derived from three independent biological experiments. All measurements were normalized to total protein content to account for differences in cell number. Secretion index analysis was performed as an additional exploratory parameter to assess secretion efficiency under treatment conditions.

### 4.2. Glucose and Lactate Assessment

Intracellular glucose levels were determined in cell lysates using an enzymatic colorimetric assay based on the hexokinase/glucose-6-phosphate dehydrogenase (HK/G6PD) reaction, performed on a Roche Cobas C111 analyzer (Roche Diagnostics, Basel, Switzerland; assay ID: 04657527190). In this method, glucose is phosphorylated by hexokinase and subsequently oxidized by glucose-6-phosphate dehydrogenase, resulting in the formation of NADPH. The generated NADPH is quantified spectrophotometrically, and its concentration is directly proportional to glucose levels in the sample. It should be noted that this assay measures glucose concentration and does not directly reflect glucose uptake or metabolic consumption.

Lactate concentration in conditioned medium was measured using a colorimetric assay (Lactate Assay Kit, MAK017, Sigma-Aldrich, Merck KGaA, Darmstadt, Germany), according to the manufacturer’s instructions. The assay is based on enzymatic conversion of lactate to a detectable product, which is quantified spectrophotometrically.

Conditioned medium samples were collected after treatment, cleared by centrifugation to remove cellular debris, and analyzed immediately or stored at appropriate conditions prior to measurement. Absorbance was measured using a microplate reader, and lactate concentrations were calculated based on a standard curve generated using the provided standards.

Results were expressed in mg/dL. Glucose and lactate levels were used for comparative analysis between treatment conditions and for calculation of the relative metabolic index (RMI).

### 4.3. Relative Metabolic Index

To assess the relationship between glucose availability and lactate production, a relative metabolic index was calculated. RMI was defined as the ratio of lactate concentration measured in conditioned medium to intracellular glucose levels obtained from cell lysates.

RMI = (lactate (medium)/glucose (intracellular))

For each experimental condition, the RMI value was normalized to the corresponding control (KN), which was set to 1.00. This normalization allowed for comparison of relative changes in metabolic activity across different treatments and cell lines.

RMI [normalized] = (RMI [treatment]/RMI [KN])

Importantly, RMI does not represent direct glycolytic flux, glucose uptake, or lactate production rate. Instead, it provides a relative measure of the balance between intracellular glucose availability and extracellular lactate levels, reflecting the overall metabolic state of the cells under the tested conditions. Higher RMI values indicate potential increased glycolytic activity.

### 4.4. Ki-67 Proliferation Index

Ki-67 levels were measured in cell lysates using a commercially available ELISA kit, according to the manufacturer’s instructions (SEC047Hu, Cloud Clone, Wuhan, China). Ki-67 index was used as a relative measure of proliferative activity and does not reflect absolute cell counts.

Cell proliferation was assessed by measuring Ki-67 levels as a marker of proliferative activity. Ki-67 values were obtained from cell lysates following treatment under the defined experimental conditions. For each experimental condition, Ki-67 values were normalized to the corresponding control (KN), which was set to 1.00. This normalization enabled comparison of relative changes in proliferative activity across different treatments and cell lines.

Ki-67 is a nuclear protein expressed during all active phases of the cell cycle and absent in resting (G0) cells; therefore, the normalized Ki-67 index reflects relative differences in cell proliferation rather than absolute proliferation rates.

### 4.5. Protein Quantification for Result Normalization

Total protein content in cell lysates was determined using a NanoDrop microvolume spectrophotometer (Thermofisher, Waltham, MA, USA) based on absorbance measurement at 280 nm. Cells were lysed in RIPA buffer supplemented with protease inhibitors, followed by centrifugation to remove insoluble material. The resulting supernatants were used for subsequent analyses. For each sample, a small volume (1–2 µL) was applied directly onto the NanoDrop measurement pedestal, and protein concentration was calculated using the instrument’s built-in quantification algorithm according to the manufacturer’s settings. Protein content was used for normalization of all measured parameters, including intracellular glucose levels, lactate concentration, and Ki-67 values, in order to account for differences in cell number and sample loading. This approach enables reliable comparison of relative changes between experimental conditions rather than absolute quantification.

The NanoDrop method was selected due to its rapid execution, minimal sample consumption, and high reproducibility. All samples were processed under identical conditions, ensuring consistency and comparability of the results.

### 4.6. Measurement of Inflammation

Inflammatory profiling was performed by quantifying a panel of cytokines and growth factors in both cell lysates (intracellular fraction) and conditioned medium (secreted fraction). The analyzed markers included: MIP-3β (macrophage inflammatory protein 3 beta, CCL19), PD-L1 (programmed death-ligand 1), MIP-1α (macrophage inflammatory protein 1 alpha, CCL3), CD40L (CD40 ligand, CD154), GROβ, IL-8, EGF, FLT (FMS-like tyrosine kinase 3 ligand), G-CSF (granulocyte colony-stimulating factor), granzyme B, IFN-β (interferon beta), IL-17E (interleukin 17E, IL-25), IL-9, IL-3, IL-33, PDGF-AA, PDGF-AB/BB, TGF-α, TRAIL (TNF-related apoptosis-inducing ligand), VEGF, and FGF.

Cytokine and growth factor concentrations were measured using a multiplex bead-based immunoassay (Luminex xMAP technology, X200-XPONRUO, Bio-Techne, Minneapolis, MN, USA) on a Luminex 200 platform, following the manufacturer’s protocol, allowing simultaneous detection of multiple analytes in a single sample. Conditioned media were collected after treatment, centrifuged to remove cellular debris, and stored under appropriate conditions until analysis. Cell lysates were prepared using standard lysis procedures. Importantly, all results were normalized to total protein content to account for differences in cell number and sample loading.

Fluorescence signals were converted into concentration values using standard curves generated for each analyte. All measurements were performed in at least three independent biological replicates and analyzed comparatively across experimental conditions to evaluate changes in inflammatory and angiogenic signalling.

### 4.7. Statistical Analysis

Due to the small sample size and non-normal data distribution, statistical analyses were performed using non-parametric methods. Differences between treatment conditions (KN, VDT, SGLT2 and DPP inhibitors) within each cell line were assessed using the Mann–Whitney U test (two-tailed). All experiments were performed in three independent biological replicates. Within each independent experiment, measurements were obtained in technical triplicates, and the mean of these technical replicates was used as one biological replicate for further analysis. Final data are presented as median values derived from three independent biological experiments. To account for multiple comparisons, false discovery rate (FDR) correction was applied using the Benjamini–Hochberg procedure, and adjusted *p*-values (q-values) were reported. Statistical significance was defined as *p* < 0.05 and q < 0.05. All analyses were performed using GraphPad Prism (version 9.5.1; GraphPad Software, Inc., San Diego, CA, USA). Data are presented as median values derived from at least three independent biological replicates. Due to the limited number of biological replicates (n = 3), the analyses should be interpreted as exploratory, and nominally significant findings should be interpreted with caution unless retained after FDR correction.

## 5. Conclusions

This study demonstrates that PTC cell lines exhibit distinct functional profiles characterized by differential regulation of angiogenesis, inflammatory signaling, and secretion efficiency. Rather than identifying a novel signaling pathway, the present study provides an integrated functional characterization of inflammatory, angiogenic, metabolic, and proliferative responses in well-established PTC models. The observed dissociation between metabolic activity and proliferation suggests that these processes may not always remain tightly coupled, highlighting a potential adaptive tumor behavior. MDA-T32 cells display a predominantly pro-angiogenic profile driven by VEGF signaling, whereas SCC147 cells exhibit a shift toward an inflammatory, mostly non-angiogenic profile, with impaired VEGF secretion despite preserved intracellular levels. Nthy-ori cells maintain a balanced and regulated cytokine profile. Targeted inhibition using VDT effectively suppressed VEGF expression and secretion in MDA-T32 cells while simultaneously enhancing IL-8 and GROβ release, indicating a shift from angiogenic to inflammatory signaling. In contrast, SGLT2 and DPP inhibition did not produce statistically significant effects after FDR correction in any cell line, although nominal changes were observed for selected markers, suggesting limited short-term efficacy of these interventions in modulating cytokine secretion. Importantly, integration of metabolic and proliferative data revealed a dissociation between glycolytic activity and cell proliferation. In MDA-T32 cells, reduced RMI following VDT treatment was accompanied by increased Ki-67 expression, indicating preserved proliferative capacity despite metabolic suppression. SCC147 cells showed minimal changes in both metabolic and proliferative parameters, reflecting limited responsiveness to treatment. In contrast, Nthy-ori cells exhibited increased metabolic activity with reduced proliferation following SGLT2 inhibition, suggesting that metabolic activation does not directly translate into enhanced cell growth in non-malignant cells. Collectively, these findings highlight metabolic plasticity as an important adaptive feature of thyroid cancer cells and suggest that modulation of metabolism alone may not be sufficient to suppress tumor growth under clinically achievable exposure conditions. Although previous studies have reported antiproliferative effects of metabolic interventions in thyroid cancer models, these responses were frequently observed at supraphysiological concentrations exceeding those typically achieved in patients.

## Figures and Tables

**Table 1:** Integrated metabolic–proliferative profile (RMI and Ki-67 index) under experimental conditions.

Cell Line	Condition	RMI (Lactate/Glucose)	Ki-67 Index
MDA-T32	KN	1.00	1.00
	VDT	0.49 ↓↓↓	1.17 ↑
	iSGLT2	0.99 ≈	1.09 ↑
	iDPP	1.00 ≈	1.17 ↑
SCC147	KN	1.00	1.00
	VDT	1.02 ≈	1.05 ≈
	iSGLT2	1.01 ≈	1.22 ↑
	iDPP	1.10 ≈	1.00 ≈
Nthy-ori	KN	1.00	1.00
	VDT	0.79 ↓	1.14 ↑
	iISGLT2	1.83 ↑↑	0.81 ↓
	iDPP	0.98 ≈	0.73 ↓

The relative metabolic index (RMI) was calculated as the ratio of lactate concentration in conditioned medium to intracellular glucose levels and normalized to control conditions (KN = 1.00). Ki-67 values were normalized to control (KN = 1.00) and used as a marker of proliferative activity. Values greater than 1 indicate increased metabolic output (RMI) or proliferation (Ki-67), whereas values lower than 1 indicate reduced activity. Arrows indicate the direction and magnitude of change relative to control: ↑ increase, ↑↑ marked increase, ↓ decrease, ↓↓↓ marked decrease, ≈ no substantial change. These annotations reflect observed differences and do not represent statistical significance. Values are presented as median index derived from three independent experiments (n = 3) each performed in technical triplicate (three technical replicates per experiment).

**Table 2:** Effects of therapeutic interventions on intracellular cytokine and growth factor levels in MDA-T32 cells (lysates).

Marker	Control (KN)	VDT	FC	*p*-Value	q-Value	iSGLT2	FC	*p*-Value	q-Value	iDPP	FC	*p*-Value	q-Value
MIP-3β	560	527	0.94	0.49	ns	527	0.94	0.49	ns	510	0.91	0.31	ns
PD-L1	2523	1961	0.78	0.03	0.08	2431	0.96	0.31	ns	2430	0.96	0.34	ns
MIP-1α	602	603	1.00	0.91	ns	576	0.96	0.37	ns	564	0.94	0.29	ns
CD40L	662	623	0.94	0.28	ns	607	0.92	0.18	ns	617	0.93	0.21	ns
GROβ	577	767	1.33	0.02	0.07	540	0.94	0.39	ns	537	0.93	0.34	ns
IL-8	587	719	1.22	0.03	0.08	616	1.05	0.41	ns	567	0.97	0.48	ns
EGF	520	498	0.96	0.42	ns	508	0.98	0.47	ns	483	0.93	0.25	ns
FLT	369	368	1.00	0.96	ns	363	0.98	0.52	ns	347	0.94	0.33	ns
G-CSF	155	192	1.24	0.07	ns	151	0.97	0.44	ns	144	0.93	0.31	ns
Granzyme B	553	541	0.98	0.66	ns	532	0.96	0.48	ns	508	0.92	0.28	ns
VEGF	2216	888	0.40	<0.001	<0.001	2240	1.01	0.89	ns	2280	1.03	0.77	ns
FGF	4680	4004	0.86	0.02	0.06	4659	1.00	0.92	ns	4741	1.01	0.84	ns

Protein levels were measured in cell lysates (intracellular fraction) following treatment with vandetanib (VDT), ISGLT2 inhibitor (iISGLT2), and IDPP inhibitor, and compared to untreated control cells (KN). Values are presented as median concentrations derived from three independent biological experiments (n = 3). Fold change (FC) represents the ratio of values in treated cells relative to control (KN). Statistical significance was assessed using the Mann–Whitney U test (two-tailed). To correct for multiple comparisons, false discovery rate (FDR) adjustment was applied using the Benjamini–Hochberg method. Differences were considered statistically significant at *p* < 0.05 and q < 0.05. “ns” indicates non-significant differences.

**Table 3:** Effects of therapeutic interventions on secreted cytokine and growth factor levels in MDA-T32 cells (conditioned medium).

Marker	Control (KN)	VDT	FC	*p*-Value	q-Value	iSGLT2	FC	*p*-Value	q-Value	iDPP	FC	*p*-Value	q-Value
MIP-3β	29.7	28.0	0.94	0.41	ns	28.7	0.97	0.52	ns	28.0	0.94	0.39	ns
PD-L1	58.7	56.0	0.95	0.36	ns	59.0	1.01	0.92	ns	57.7	0.98	0.61	ns
MIP-1α	32.8	31.3	0.95	0.44	ns	33.5	1.02	0.78	ns	31.7	0.97	0.52	ns
CD40L	49.3	55.7	1.13	0.12	ns	51.3	1.04	0.49	ns	51.0	1.03	0.57	ns
GROβ	330.0	787.0	2.38	0.008	0.03	322.0	0.98	0.66	ns	275.0	0.83	0.09	ns
IL-8	4130.0	5992.0	1.45	0.01	0.04	4047.0	0.98	0.71	ns	3389.0	0.82	0.03	0.09
EGF	51.0	52.3	1.03	0.83	ns	51.3	1.01	0.92	ns	49.0	0.96	0.54	ns
FLT	75.3	82.5	1.10	0.17	ns	75.7	1.01	0.88	ns	69.0	0.92	0.22	ns
G-CSF	42.5	34.3	0.81	0.09	ns	42.7	1.00	0.94	ns	41.7	0.98	0.83	ns
Granzyme B	28.0	25.2	0.90	0.28	ns	29.3	1.05	0.44	ns	27.3	0.98	0.67	ns
VEGF	5310.0	1310.0	0.25	<0.001	<0.001	5258.0	0.99	0.91	ns	5497.0	1.04	0.62	ns
FGF	23.7	27.3	1.15	0.14	ns	23.0	0.97	0.61	ns	23.2	0.98	0.73	ns

Protein levels were measured in conditioned medium (secreted fraction) following treatment with vandetanib (VDT), ISGLT2 inhibitor (iISGLT2), and IDPP inhibitor, and compared to untreated control cells (KN). Values are presented as median concentrations derived from three independent biological experiments (n = 3), each performed in technical triplicate (three technical replicates per experiment). Fold change (FC) represents the ratio of values in treated cells relative to control (KN). Statistical significance was assessed using the Mann–Whitney U test (two-tailed). To correct for multiple comparisons, false discovery rate (FDR) adjustment was applied using the Benjamini–Hochberg method. Differences were considered statistically significant at *p* < 0.05 and q < 0.05. “ns” indicates non-significant differences.

**Table 4:** Effects of therapeutic interventions on secretion efficiency (index) in MDA-T32 cells.

Marker	Control (KN)	VDT	FC	*p*-Value	q-Value	iSGLT2	FC	*p*-Value	q-Value	iDPP	FC	*p*-Value	q-Value
MIP-3β	0.053	0.054	1.02	0.400	ns	0.052	0.99	1.000	ns	0.045	0.85	0.700	ns
PD-L1	0.023	0.028	1.22	0.100	ns	0.016	0.69	0.400	ns	0.020	0.87	1.000	ns
MIP-1α	0.056	0.052	0.93	0.700	ns	0.089	1.60	0.400	ns	0.059	1.07	0.700	ns
CD40L	0.075	0.088	1.17	0.100	ns	0.058	0.78	0.200	ns	0.066	0.89	0.400	ns
GROβ	0.573	1.022	1.78	0.100	ns	0.594	1.04	0.700	ns	0.544	0.95	0.400	ns
IL-8	7.020	8.265	1.18	0.100	ns	6.552	0.93	0.700	ns	6.005	0.86	0.100	ns
EGF	0.098	0.104	1.06	1.000	ns	0.101	1.03	1.000	ns	0.089	0.91	1.000	ns
FLT	0.206	0.224	1.09	0.700	ns	0.207	1.01	0.700	ns	0.199	0.97	1.000	ns
G-CSF	0.275	0.175	0.63	0.100	ns	0.281	1.02	1.000	ns	0.291	1.06	0.400	ns
Granzyme B	0.051	0.046	0.89	0.100	ns	0.055	1.08	0.700	ns	0.056	1.09	0.700	ns
IFN-β	0.577	0.540	0.94	0.700	ns	0.616	1.07	0.658	ns	0.579	1.00	1.000	ns
IL-17E	0.588	0.522	0.89	0.100	ns	0.521	0.89	0.200	ns	0.572	0.97	1.000	ns
IL-9	0.021	0.020	0.97	1.000	ns	0.023	1.08	1.000	ns	0.022	1.05	0.200	ns
IL-3	0.056	0.055	0.98	1.000	ns	0.056	0.99	0.700	ns	0.058	1.04	0.200	ns
IL-33	0.046	0.042	0.92	1.000	ns	0.046	1.00	1.000	ns	0.049	1.06	0.700	ns
PDGF-AA	1.003	0.679	0.68	0.100	ns	0.945	0.94	1.000	ns	1.052	1.05	0.400	ns
PDGF-AB/BB	0.220	0.196	0.89	0.700	ns	0.227	1.03	1.000	ns	0.235	1.07	0.400	ns
TGF-α	0.215	0.065	0.30	0.400	ns	0.157	0.73	1.000	ns	0.227	1.05	0.400	ns
TRAIL	0.092	0.076	0.82	1.000	ns	0.099	1.08	0.700	ns	0.098	1.06	1.000	ns
VEGF	2.386	1.466	0.61	0.100	ns	2.355	0.99	1.000	ns	2.396	1.00	1.000	ns
FGF	0.0050	0.0069	1.38	0.100	ns	0.0049	0.97	1.000	ns	0.0049	0.98	1.000	ns

The secretion index was calculated as the ratio of protein concentration in conditioned medium to that in corresponding cell lysates (medium/lysate) for each independent experiment. Values are presented as median index values derived from three independent biological experiments (n = 3), each performed in technical triplicate (three technical replicates per experiment). Fold change (FC) represents the ratio of index values in treated cells relative to untreated control cells (KN). Statistical significance was assessed using the Mann–Whitney U test (two-tailed). To correct for multiple comparisons, false discovery rate (FDR) adjustment was applied using the Benjamini–Hochberg method. No statistically significant differences were observed after FDR correction. “ns” indicates non-significant differences.

**Table 5:** Effects of therapeutic interventions on intracellular cytokine and growth factor levels in SCC147 cells (lysates).

Marker	Control (KN)	VDT	FC	*p*-Value	q-Value	iSGLT2	FC	*p*-Value	q-Value	iDPP	FC	*p*-Value	q-Value
MIP-3β	685	654	0.95	0.31	ns	683	1.00	0.84	ns	605	0.88	0.22	ns
PD-L1	2988	3140	1.05	0.41	ns	2995	1.00	0.92	ns	3118	1.04	0.38	ns
MIP-1α	914	877	0.96	0.36	ns	909	0.99	0.88	ns	806	0.88	0.19	ns
CD40L	818	792	0.97	0.44	ns	828	1.01	0.91	ns	738	0.90	0.21	ns
GROβ	885	824	0.93	0.33	ns	858	0.97	0.47	ns	758	0.86	0.18	ns
IL-8	1888	1716	0.91	0.12	ns	1885	1.00	0.95	ns	1832	0.97	0.54	ns
EGF	706	675	0.96	0.41	ns	712	1.01	0.89	ns	619	0.88	0.19	ns
FLT	560	528	0.94	0.37	ns	561	1.00	0.96	ns	499	0.89	0.23	ns
G-CSF	284	271	0.95	0.44	ns	279	0.98	0.71	ns	231	0.81	0.15	ns
Granzyme B	732	706	0.96	0.52	ns	745	1.02	0.81	ns	662	0.90	0.28	ns
VEGF	664	627	0.94	0.28	ns	676	1.02	0.76	ns	603	0.91	0.21	ns
FGF	1952	2042	1.05	0.47	ns	2002	1.03	0.66	ns	1941	0.99	0.93	ns

Protein levels were measured in cell lysates (intracellular fraction) following treatment with vandetanib (VDT), ISGLT2 inhibitor (iISGLT2), and IDPP inhibitor, and compared to untreated control cells (KN). Values are presented as median concentrations derived from three independent biological experiments (n = 3) each performed in technical triplicate (three technical replicates per experiment). Fold change (FC) represents the ratio of values in treated cells relative to control (KN). Statistical significance was assessed using the Mann–Whitney U test (two-tailed). To correct for multiple comparisons, false discovery rate (FDR) adjustment was applied using the Benjamini–Hochberg method. Differences were considered statistically significant at *p* < 0.05 and q < 0.05. “ns” indicates non-significant differences. No statistically significant differences were observed after FDR correction.

**Table 6:** Effects of therapeutic interventions on secreted cytokine and growth factor levels in SCC147 cells (conditioned medium).

Marker	Control (KN)	VDT	FC	*p*-Value	q-Value	iSGLT2	FC	*p*-Value	q-Value	iDPP	FC	*p*-Value	q-Value
MIP-3β	34.3	32.7	0.95	0.39	ns	34.7	1.01	0.87	ns	33.0	0.96	0.62	ns
PD-L1	118.5	110.2	0.93	0.21	ns	120.2	1.01	0.92	ns	115.5	0.97	0.66	ns
MIP-1α	36.0	34.5	0.96	0.41	ns	35.3	0.98	0.71	ns	35.0	0.97	0.62	ns
CD40L	55.0	51.5	0.94	0.33	ns	54.7	0.99	0.84	ns	52.3	0.95	0.48	ns
GROβ	170.2	130.3	0.77	0.12	ns	212.3	1.25	0.18	ns	132.3	0.78	0.11	ns
IL-8	10,863.0	10,036.0	0.92	0.17	ns	11,565.0	1.06	0.31	ns	10,478.0	0.96	0.42	ns
EGF	51.7	50.2	0.97	0.72	ns	52.3	1.01	0.88	ns	51.0	0.99	0.93	ns
FLT	131.2	126.5	0.96	0.48	ns	135.7	1.03	0.66	ns	130.3	0.99	0.91	ns
G-CSF	25.7	24.3	0.95	0.51	ns	25.3	0.99	0.84	ns	25.0	0.97	0.72	ns
Granzyme B	30.3	29.2	0.96	0.63	ns	31.3	1.03	0.76	ns	29.7	0.98	0.81	ns
VEGF	24.7	23.0	0.93	0.28	ns	25.0	1.01	0.91	ns	23.3	0.94	0.47	ns
FGF	68.2	55.3	0.81	0.14	ns	75.8	1.11	0.22	ns	59.0	0.87	0.19	ns

Protein levels were measured in conditioned medium (secreted fraction) following treatment with vandetanib (VDT), ISGLT2 inhibitor (iISGLT2), and IDPP inhibitor, and compared to untreated control cells (KN). Values are presented as median concentrations derived from three independent biological experiments (n = 3) each performed in technical triplicate (three technical replicates per experiment). Fold change (FC) represents the ratio of values in treated cells relative to control (KN). Statistical significance was assessed using the Mann–Whitney U test (two-tailed). To correct for multiple comparisons, false discovery rate (FDR) adjustment was applied using the Benjamini–Hochberg method. Differences were considered statistically significant at *p* < 0.05 and q < 0.05. “ns” indicates non-significant differences. No statistically significant differences were observed after FDR correction.

**Table 7:** Effects of therapeutic interventions on secretion efficiency (index) in SCC147 cells.

Marker	Control (KN)	VDT	FC	*p*-Value	q-Value	iSGLT2	FC	*p*-Value	q-Value	iDPP	FC	*p*-Value	q-Value
MIP-3β	0.050	0.050	1.00	0.91	ns	0.051	1.02	0.84	ns	0.055	1.10	0.62	ns
PD-L1	0.040	0.035	0.87	0.41	ns	0.040	1.00	0.91	ns	0.037	0.93	0.66	ns
MIP-1α	0.039	0.039	1.00	0.88	ns	0.039	1.00	0.92	ns	0.043	1.10	0.61	ns
CD40L	0.067	0.065	0.97	0.73	ns	0.066	0.99	0.84	ns	0.071	1.06	0.48	ns
GROβ	0.192	0.158	0.82	0.22	ns	0.247	1.29	0.18	ns	0.174	0.91	0.47	ns
IL-8	5.75	5.85	1.02	0.79	ns	6.14	1.07	0.31	ns	5.72	1.00	0.92	ns
EGF	0.073	0.074	1.01	0.91	ns	0.073	1.00	0.94	ns	0.082	1.12	0.52	ns
FLT	0.235	0.239	1.02	0.88	ns	0.242	1.03	0.76	ns	0.261	1.11	0.44	ns
G-CSF	0.090	0.090	1.00	0.95	ns	0.091	1.01	0.88	ns	0.108	1.20	0.39	ns
Granzyme B	0.041	0.041	1.00	0.91	ns	0.042	1.02	0.81	ns	0.045	1.10	0.58	ns
VEGF	0.037	0.037	1.00	0.94	ns	0.037	1.00	0.91	ns	0.039	1.05	0.47	ns
FGF	0.035	0.027	0.77	0.18	ns	0.038	1.08	0.22	ns	0.030	0.85	0.19	ns

The secretion index was calculated as the ratio of protein concentration in conditioned medium to that in corresponding cell lysates (medium/lysate) for each independent experiment. Values are presented as median index values derived from three independent biological experiments (n = 3) each performed in technical triplicate (three technical replicates per experiment). Fold change (FC) represents the ratio of index values in treated cells relative to untreated control cells (KN). Statistical significance was assessed using the Mann–Whitney U test (two-tailed). To correct for multiple comparisons, false discovery rate (FDR) adjustment was applied using the Benjamini–Hochberg method. No statistically significant differences were observed after FDR correction. “ns” indicates non-significant differences.

**Table 8:** Effects of therapeutic interventions on intracellular cytokine and growth factor levels in Nthy-ori cells (lysates).

Marker	Control (KN)	VDT	FC	*p*-Value	q-Value	iSGLT2	FC	*p*-Value	q-Value	iDPP	FC	*p*-Value	q-Value
MIP-3β	642	559	0.87	0.09	ns	520	0.81	0.06	ns	558	0.87	0.11	ns
PD-L1	2677	2377	0.89	0.14	ns	2636	0.98	0.71	ns	2478	0.93	0.33	ns
MIP-1α	800	696	0.87	0.08	ns	635	0.79	0.06	ns	702	0.88	0.12	ns
CD40L	753	665	0.88	0.11	ns	622	0.83	0.07	ns	666	0.88	0.14	ns
GROβ	749	650	0.87	0.10	ns	536	0.72	0.05	ns	588	0.79	0.06	ns
IL-8	2680	983	0.37	0.02	0.09	1160	0.43	0.03	0.11	1120	0.42	0.03	0.11
EGF	634	555	0.88	0.12	ns	509	0.80	0.07	ns	546	0.86	0.15	ns
FLT	507	400	0.79	0.08	ns	374	0.74	0.06	ns	415	0.82	0.09	ns
G-CSF	226	201	0.89	0.18	ns	156	0.69	0.06	ns	191	0.85	0.11	ns
Granzyme B	668	572	0.86	0.21	ns	529	0.79	0.12	ns	574	0.86	0.19	ns
IFN-β	28.0	25.5	0.91	0.33	ns	22.0	0.79	0.09	ns	25.5	0.91	0.28	ns
IL-17E	27.8	29.0	1.04	0.61	ns	30.1	1.08	0.48	ns	29.6	1.06	0.53	ns
IL-9	516	559	1.08	0.41	ns	502	0.97	0.68	ns	503	0.97	0.64	ns
IL-3	539	457	0.85	0.07	ns	437	0.81	0.06	ns	468	0.87	0.11	ns
IL-33	490	436	0.89	0.18	ns	375	0.77	0.07	ns	408	0.83	0.09	ns
PDGF-AA	468	385	0.82	0.09	ns	369	0.79	0.07	ns	397	0.85	0.12	ns
PDGF-AB/BB	261	220	0.84	0.11	ns	224	0.86	0.14	ns	236	0.90	0.21	ns
TGF-α	312	280	0.90	0.16	ns	243	0.78	0.08	ns	276	0.88	0.13	ns
TRAIL	517	470	0.91	0.19	ns	346	0.67	0.06	ns	442	0.85	0.12	ns
VEGF	708	554	0.78	0.03	0.10	563	0.80	0.05	ns	603	0.85	0.09	ns
FGF	1454	1318	0.91	0.04	0.12	1409	0.97	0.62	ns	1382	0.95	0.48	ns

Protein levels were measured in cell lysates (intracellular fraction) following treatment with vandetanib (VDT), ISGLT2 inhibitor (iISGLT2), and IDPP inhibitor, and compared to untreated control cells (KN). Values are presented as median concentrations derived from three independent biological experiments (n = 3), each performed in technical triplicate (three technical replicates per experiment). Fold change (FC) represents the ratio of values in treated cells relative to control (KN). Statistical significance was assessed using the Mann–Whitney U test (two-tailed). To correct for multiple comparisons, false discovery rate (FDR) adjustment was applied using the Benjamini–Hochberg method. Although selected markers (e.g., IL-8, VEGF, and FGF) reached nominal statistical significance (*p* < 0.05), none of the observed differences remained significant after FDR correction. “ns” indicates non-significant differences.

**Table 9:** Effects of therapeutic interventions on cytokine and growth factor levels in Nthy-ori cells (conditioned medium).

Marker	Control (KN)	VDT	FC	*p*-Value	q-Value	iSGLT2	FC	*p*-Value	q-Value	iDPP	FC	*p*-Value	q-Value
MIP-3β	642	559	0.87	0.09	ns	520	0.81	0.06	ns	558	0.87	0.11	ns
PD-L1	2677	2377	0.89	0.14	ns	2636	0.98	0.71	ns	2478	0.93	0.33	ns
MIP-1α	800	696	0.87	0.08	ns	635	0.79	0.06	ns	702	0.88	0.12	ns
CD40L	753	665	0.88	0.11	ns	622	0.83	0.07	ns	666	0.88	0.14	ns
GROβ	749	650	0.87	0.10	ns	536	0.72	0.05	ns	588	0.79	0.06	ns
IL-8	2680	983	0.37	0.02	0.09	1160	0.43	0.03	0.11	1120	0.42	0.03	0.11
EGF	634	555	0.88	0.12	ns	509	0.80	0.07	ns	546	0.86	0.15	ns
FLT	507	400	0.79	0.08	ns	374	0.74	0.06	ns	415	0.82	0.09	ns
G-CSF	226	201	0.89	0.18	ns	156	0.69	0.06	ns	191	0.85	0.11	ns
Granzyme B	668	572	0.86	0.21	ns	529	0.79	0.12	ns	574	0.86	0.19	ns
IFN-β	28.0	25.5	0.91	0.33	ns	22.0	0.79	0.09	ns	25.5	0.91	0.28	ns
IL-17E	27.8	29.0	1.04	0.61	ns	30.1	1.08	0.48	ns	29.6	1.06	0.53	ns
IL-9	516	559	1.08	0.41	ns	502	0.97	0.68	ns	503	0.97	0.64	ns
IL-3	539	457	0.85	0.07	ns	437	0.81	0.06	ns	468	0.87	0.11	ns
IL-33	490	436	0.89	0.18	ns	375	0.77	0.07	ns	408	0.83	0.09	ns
PDGF-AA	468	385	0.82	0.09	ns	369	0.79	0.07	ns	397	0.85	0.12	ns
PDGF-AB/BB	261	220	0.84	0.11	ns	224	0.86	0.14	ns	236	0.90	0.21	ns
TGF-α	312	280	0.90	0.16	ns	243	0.78	0.08	ns	276	0.88	0.13	ns
TRAIL	517	470	0.91	0.19	ns	346	0.67	0.06	ns	442	0.85	0.12	ns
VEGF	708	554	0.78	0.03	0.10	563	0.80	0.05	ns	603	0.85	0.09	ns
FGF	1454	1318	0.91	0.04	0.12	1409	0.97	0.62	ns	1382	0.95	0.48	ns

Protein levels were measured in conditioned medium (secreted fraction) following treatment with vandetanib (VDT), ISGLT2 inhibitor (iISGLT2), and IDPP inhibitor, and compared to untreated control cells (KN). Values are presented as median concentrations derived from three independent biological experiments (n = 3), each performed in technical triplicate (three technical replicates per experiment). Fold change (FC) represents the ratio of values in treated cells relative to control (KN). Statistical significance was assessed using the Mann–Whitney U test (two-tailed). To correct for multiple comparisons, false discovery rate (FDR) adjustment was applied using the Benjamini–Hochberg method. Although several markers (including IL-8, VEGF, and FGF) reached nominal statistical significance (*p* < 0.05), none of the observed differences remained significant after FDR correction. “ns” indicates non-significant differences.

**Table 10:** Effects of therapeutic interventions on secretion efficiency (index) in Nthy-ori cells.

Marker	KN	VDT	FC (VDT/KN)	iSGLT2	FC	iDPP	FC
MIP3B	0.060	0.052	0.87	0.073	1.22	0.063	1.05
PD-L1/B7-HI	0.040	0.033	0.83	0.038	0.95	0.041	1.03
MIP1A	0.048	0.047	0.98	0.059	1.23	0.054	1.13
CD40L	0.084	0.074	0.88	0.093	1.11	0.086	1.02
GROB	0.567	0.349	0.62	0.643	1.13	0.512	0.90
IL8	4.540	7.760	1.71	10.127	2.23	9.511	2.10
EGF	0.083	0.089	1.07	0.103	1.24	0.097	1.17
FLT	0.314	0.241	0.77	0.391	1.25	0.321	1.02
G-CSF	0.128	0.129	1.01	0.186	1.45	0.152	1.19
Granzyme B	0.051	0.044	0.86	0.064	1.25	0.058	1.14
INFB	0.436	0.462	1.06	0.545	1.25	0.471	1.08
IL17E	0.545	0.479	0.88	0.448	0.82	0.433	0.79
IL9	0.027	0.023	0.85	0.027	1.00	0.026	0.96
IL3	0.056	0.053	0.95	0.070	1.25	0.062	1.11
IL33	0.045	0.039	0.87	0.055	1.22	0.045	1.00
PDGFAA	0.954	0.788	0.83	1.209	1.27	1.224	1.28
PDGF-AB/BB	0.138	0.172	1.25	0.157	1.14	0.153	1.11
TGFA	0.049	0.048	0.98	0.069	1.41	0.056	1.14
TRAIL	0.071	0.054	0.76	0.093	1.31	0.069	0.97
VEGF	0.825	0.570	0.69	0.956	1.16	0.901	1.09
FGF	0.018	0.019	1.06	0.018	1.00	0.020	1.11

The secretion index was calculated as the ratio of protein concentration in conditioned medium to that in the corresponding cell lysates (medium/lysate) for each independent experiment. Values are presented as median indices derived from three independent biological experiments (n = 3), each performed in technical triplicate (three technical replicates per experiment). Fold change (FC) represents the ratio of index values in treated cells relative to untreated control cells (KN), where FC > 1 indicates increased secretion efficiency and FC < 1 indicates reduced secretion efficiency. Statistical significance was assessed using the Mann–Whitney U test (two-tailed). To correct for multiple comparisons, false discovery rate (FDR) adjustment was applied using the Benjamini–Hochberg method. Differences were considered statistically significant at *p* < 0.05 and q (FDR-adjusted *p*-value) < 0.05. No statistically significant differences were observed after FDR correction.

## Data Availability

The datasets analyzed during the current study are available from the corresponding author upon reasonable request.

## Supplementary Materials

The following supporting information can be downloaded at: https://www.mdpi.com/article/10.3390/ijms27146131/s1.
