# Supplementary material for: Integrated Cytokine, Metabolic, and Proliferative Profiling Reveals Divergent Metabolic and Proliferative Responses in Papillary Thyroid Cancer Cells
Source: Int J Mol Sci. 2026 Jul 9;27(14):6131. doi: 10.3390/ijms27146131 (PMC13411649; doi:10.3390/ijms27146131)
Supplement: Supplementary file 1 [file ijms-27-06131-s001.zip › ijms-4334031-supplementary.pdf]

Supplementary file

Supplementary Table S1. MDA-T32 vs Nthy-ori – cytokine and growth factor levels.

Marker	MDA-T32 Lysate (median)	Control Lysate (median)	FC	p-value	q-value	MDA-T32 Medium (median)	Control Medium (median)	FC	p-value	q-value
MIP-3β	560.1	642.0	0.87	0.31	ns	29.7	41.3	0.72	0.20	ns
PD-L1	2522.9	2677.4	0.94	0.42	ns	58.7	115.2	0.51	0.18	ns
MIP-1α	601.6	799.5	0.75	0.28	ns	32.8	41.0	0.80	0.25	ns
CD40L	661.6	752.4	0.88	0.37	ns	49.3	69.3	0.71	0.22	ns
GROβ	577.4	749.4	0.77	0.26	ns	330.3	445.7	0.74	0.41	ns
IL-8	587.0	2678.4	0.22	0.004	0.01	4129.8	12325.7	0.33	0.003	0.009
EGF	519.8	633.5	0.82	0.30	ns	51.0	62.0	0.82	0.33	ns
FLT	368.6	506.6	0.73	0.21	ns	75.3	171.8	0.44	0.09	ns
G-CSF	155.0	226.3	0.68	0.19	ns	42.5	30.7	1.38	0.27	ns
Granzyme B	553.4	667.5	0.83	0.34	ns	28.0	37.7	0.74	0.29	ns
IFN-β	19.8	27.8	0.71	0.23	ns	11.3	12.0	0.94	0.44	ns
IL-17E	38.0	27.8	1.37	0.15	ns	22.3	15.0	1.49	0.17	ns
IL-9	647.5	516.1	1.25	0.18	ns	13.7	14.7	0.93	0.39	ns
IL-3	455.9	539.6	0.85	0.36	ns	25.3	32.3	0.78	0.31	ns
IL-33	371.0	490.1	0.76	0.27	ns	16.7	23.0	0.73	0.28	ns
PDGF-AA	391.1	468.4	0.84	0.33	ns	389.0	482.5	0.81	0.35	ns
PDGF-AB/BB	167.6	260.8	0.64	0.12	ns	36.7	39.3	0.93	0.40	ns
TGF-α	215.1	311.6	0.69	0.20	ns	45.7	16.0	2.85	0.48	ns
TRAIL	338.1	517.1	0.65	0.14	ns	31.0	36.5	0.85	0.32	ns
VEGF	2216.1	707.9	3.13	<0.001	<0.001	5310.0	628.7	8.45	<0.001	<0.001
FGF	4679.5	1453.8	3.22	<0.001	<0.001	23.7	29.0	0.82	0.36	ns

Protein levels were measured in cell lysates (intracellular fraction) and conditioned medium (secreted fraction). Values are presented as median concentrations derived from three independent biological experiments (n = 3) each performed in technical triplicate (three technical replicates per experiment). Fold change (FC) represents the ratio of MDA-T32 to control values, calculated separately for lysates and conditioned medium. Statistical significance was assessed using the Mann–Whitney U test (two-tailed). To correct for multiple comparisons, false discovery rate (FDR) adjustment was applied using the Benjamini–Hochberg method. Differences were considered statistically significant at $p < 0.05$ and $q < 0.05$. "ns" indicates non-significant differences.

Supplementary Table S2. MDA-T32 vs Nthy-ori – secretion index.

Marker	MDA-T32 Index (median)	Control Index (median)	FC	p-value	q-value
MIP-3β	0.05	0.06	0.83	0.28	ns
PD-L1	0.02	0.04	0.50	0.12	ns
MIP-1α	0.05	0.05	1.00	0.44	ns
CD40L	0.07	0.09	0.78	0.30	ns
GROβ	0.57	0.59	0.97	0.41	ns
IL-8	7.04	4.60	1.53	0.006	0.02
EGF	0.10	0.10	1.00	0.48	ns
FLT	0.20	0.34	0.59	0.09	ns
G-CSF	0.27	0.14	1.93	0.11	ns
Granzyme B	0.05	0.06	0.83	0.33	ns
IFN-β	0.57	0.43	1.33	0.21	ns
IL-17E	0.59	0.54	1.09	0.36	ns
IL-9	0.02	0.03	0.67	0.25	ns

Marker	MDA-T32 Index (median)	Control Index (median)	FC	p-value	q-value
IL-3	0.06	0.06	1.00	0.47	ns
IL-33	0.04	0.05	0.80	0.29	ns
PDGF-AA	1.00	1.03	0.97	0.42	ns
PDGF-AB/BB	0.22	0.15	1.47	0.18	ns
TGF-α	0.21	0.05	4.20	0.07	ns
TRAIL	0.09	0.07	1.29	0.22	ns
VEGF	2.39	0.89	2.69	0.002	0.008
FGF	0.005	0.02	0.25	0.004	0.01

The secretion index was calculated as the ratio of protein concentration in conditioned medium to that in corresponding cell lysates (medium/lysate). Values are presented as median index values derived from three independent biological experiments (n = 3) each performed in technical triplicate (three technical replicates per experiment). Fold change (FC) represents the ratio of index values in MDA-T32 cells relative to control cells. Statistical significance was assessed using the Mann–Whitney U test (two-tailed). To correct for multiple comparisons, false discovery rate (FDR) adjustment was applied using the Benjamini–Hochberg method. Differences were considered statistically significant at $p < 0.05$ and $q < 0.05$. "ns" indicates non-significant differences.

Supplementary Table S3. SCC147 vs Nthy-ori – cytokine and growth factor levels.

Marker	SCC147 Lysate (median)	Control Lysate (median)	FC	p-value	q-value	SCC147 Medium (median)	Control Medium (median)	FC	p-value	q-value
MIP-3β	684.6	642.0	1.07	0.38	ns	34.3	41.3	0.83	0.29	ns
PD-L1	2988.1	2677.4	1.12	0.22	ns	118.5	115.2	1.03	0.44	ns
MIP-1α	913.9	799.5	1.14	0.24	ns	36.0	41.0	0.88	0.31	ns
CD40L	818.0	752.4	1.09	0.30	ns	55.0	69.3	0.79	0.27	ns
GROβ	885.5	749.4	1.18	0.19	ns	170.2	445.7	0.38	0.01	0.04
IL-8	1888.3	2678.4	0.70	0.008	0.02	10863.2	12325.7	0.88	0.12	ns
EGF	706.3	633.5	1.11	0.33	ns	51.7	62.0	0.83	0.28	ns
FLT	559.8	506.6	1.10	0.34	ns	131.2	171.8	0.76	0.21	ns
G-CSF	283.9	226.3	1.25	0.18	ns	25.7	30.7	0.84	0.30	ns
Granzyme B	732.3	667.5	1.10	0.29	ns	30.3	37.7	0.80	0.32	ns
IFN-β	32.0	27.8	1.15	0.26	ns	11.3	12.0	0.94	0.41	ns
IL-17E	33.5	27.8	1.21	0.21	ns	10.7	15.0	0.71	0.24	ns
IL-9	513.3	516.1	1.00	0.47	ns	13.3	14.7	0.90	0.39	ns
IL-3	604.9	539.6	1.12	0.31	ns	27.3	32.3	0.85	0.33	ns
IL-33	588.9	490.1	1.20	0.22	ns	18.3	23.0	0.80	0.28	ns
PDGF-AA	469.6	468.4	1.00	0.49	ns	515.7	482.5	1.07	0.37	ns
PDGF-AB/BB	263.5	260.8	1.01	0.46	ns	35.3	39.3	0.90	0.35	ns
TGF-α	405.1	311.6	1.30	0.17	ns	15.3	16.0	0.96	0.42	ns
TRAIL	635.3	517.1	1.23	0.19	ns	25.3	36.5	0.69	0.22	ns
VEGF	664.3	707.9	0.94	0.41	ns	24.3	628.7	0.04	<0.001	<0.001
FGF	1951.9	1453.8	1.34	0.12	ns	68.2	29.0	2.35	0.02	0.05

Protein levels were measured in cell lysates (intracellular fraction) and conditioned medium (secreted fraction). Values are presented as median concentrations derived from three independent biological experiments (n = 3) each performed in technical triplicate (three technical replicates per experiment). Fold change (FC) represents the ratio of SCC147 to control values, calculated separately for lysates and conditioned medium. Statistical significance was assessed using the Mann–Whitney U test (two-tailed). To correct for multiple comparisons, false discovery rate (FDR) adjustment was applied using the Benjamini–Hochberg method. Differences were considered statistically significant at $p < 0.05$ and $q < 0.05$. "ns" indicates non-significant differences.

Supplementary Table S4. SCC147 vs Nthy-ori – secretion index.

Marker	SCC147 Index (median)	Control Index (median)	FC	p-value	q-value
MIP-3β	0.05	0.06	0.83	0.29	ns
PD-L1	0.04	0.04	1.00	0.41	ns
MIP-1α	0.04	0.05	0.80	0.27	ns
CD40L	0.07	0.09	0.78	0.31	ns
GROβ	0.19	0.59	0.32	0.008	0.02
IL-8	5.75	4.60	1.25	0.01	0.03
EGF	0.07	0.10	0.70	0.25	ns
FLT	0.23	0.34	0.68	0.19	ns
G-CSF	0.09	0.14	0.64	0.22	ns
Granzyme B	0.04	0.06	0.67	0.28	ns
IFN-β	0.35	0.43	0.81	0.30	ns
IL-17E	0.32	0.54	0.59	0.18	ns
IL-9	0.03	0.03	1.00	0.46	ns
IL-3	0.05	0.06	0.83	0.34	ns
IL-33	0.03	0.05	0.60	0.24	ns
PDGF-AA	1.10	1.03	1.07	0.39	ns
PDGF-AB/BB	0.13	0.15	0.87	0.36	ns
TGF-α	0.04	0.05	0.80	0.33	ns
TRAIL	0.04	0.07	0.57	0.21	ns
VEGF	0.04	0.89	0.05	<0.001	<0.001
FGF	0.03	0.02	1.50	0.02	0.05

The secretion index was calculated as the ratio of protein concentration in conditioned medium to that in corresponding cell lysates (medium/lysate). Values are presented as median index values derived from three independent biological experiments (n = 3) each performed in technical triplicate (three technical replicates per experiment). Fold change (FC) represents the ratio of index values in SCC147 cells relative to control cells. Statistical significance was assessed using the Mann–Whitney U test (two-tailed). To correct for multiple comparisons, false discovery rate (FDR) adjustment was applied using the Benjamini–Hochberg method. Differences were considered statistically significant at $p < 0.05$ and $q < 0.05$. "ns" indicates non-significant differences.

Supplementary Table S5. MDA-T32 vs SCC147 – cytokine and growth factor levels.

Marker	MDA-T32 Lysate (median)	SCC147 Lysate (median)	FC	p-value	q-value	MDA-T32 Medium (median)	SCC147 Medium (median)	FC	p-value	q-value
MIP-3β	560.1	684.6	0.82	0.21	ns	29.7	34.3	0.87	0.33	ns
PD-L1	2522.9	2988.1	0.84	0.19	ns	58.7	118.5	0.50	0.08	ns
MIP-1α	601.6	913.9	0.66	0.02	0.05	32.8	36.0	0.91	0.41	ns
CD40L	661.6	818.0	0.81	0.17	ns	49.3	55.0	0.90	0.38	ns
GROβ	577.4	885.5	0.65	0.02	0.05	330.3	170.2	1.94	0.01	0.03
IL-8	587.0	1888.3	0.31	<0.001	<0.001	4129.8	10863.2	0.38	<0.001	<0.001
EGF	519.8	706.3	0.74	0.23	ns	51.0	51.7	0.99	0.49	ns
FLT	368.6	559.8	0.66	0.11	ns	75.3	131.2	0.57	0.07	ns
G-CSF	155.0	283.9	0.55	0.09	ns	42.5	25.7	1.65	0.12	ns
Granzyme B	553.4	732.3	0.76	0.25	ns	28.0	30.3	0.92	0.43	ns
IFN-β	19.8	32.0	0.62	0.14	ns	11.3	11.3	1.00	0.50	ns
IL-17E	38.0	33.5	1.13	0.34	ns	22.3	10.7	2.08	0.09	ns
IL-9	647.5	513.3	1.26	0.20	ns	13.7	13.3	1.03	0.47	ns
IL-3	455.9	604.9	0.75	0.28	ns	25.3	27.3	0.93	0.40	ns
IL-33	371.0	588.9	0.63	0.13	ns	16.7	18.3	0.91	0.42	ns

Marker	MDA-T32 Lysate (median)	SCC147 Lysate (median)	FC	p-value	q-value	MDA-T32 Medium (median)	SCC147 Medium (median)	FC	p-value	q-value
PDGF-AA	391.1	469.6	0.83	0.31	ns	389.0	515.7	0.75	0.22	ns
PDGF-AB/BB	167.6	263.5	0.64	0.10	ns	36.7	35.3	1.04	0.45	ns
TGF-α	215.1	405.1	0.53	0.03	ns	45.7	15.3	2.99	0.01	0.03
TRAIL	338.1	635.3	0.53	0.08	ns	31.0	25.3	1.22	0.31	ns
VEGF	2216.1	664.3	3.33	<0.001	<0.001	5310.0	24.3	218.0	<0.001	<0.001
FGF	4679.5	1951.9	2.40	<0.001	<0.001	23.7	68.2	0.35	0.02	0.05

Protein levels were measured in cell lysates (intracellular fraction) and conditioned medium (secreted fraction). Values are presented as median concentrations derived from three independent biological experiments (n = 3) each performed in technical triplicate (three technical replicates per experiment). Fold change (FC) represents the ratio of MDA-T32 to SCC147 values, calculated separately for lysates and conditioned medium. Statistical significance was assessed using the Mann–Whitney U test (two-tailed). To correct for multiple comparisons, false discovery rate (FDR) adjustment was applied using the Benjamini–Hochberg method. Differences were considered statistically significant at $p < 0.05$ and $q < 0.05$. "ns" indicates non-significant differences.

Supplementary Table S6. MDA-T32 vs SCC147 – secretion index.

Marker	MDA-T32 Index (median)	SCC147 Index (median)	FC	p-value	q-value
MIP-3β	0.05	0.05	1.00	0.42	ns
PD-L1	0.02	0.04	0.50	0.11	ns
MIP-1α	0.05	0.04	1.25	0.19	ns
CD40L	0.07	0.07	1.00	0.47	ns
GROβ	0.57	0.19	3.00	0.008	0.02
IL-8	7.04	5.75	1.22	0.01	0.03
EGF	0.10	0.07	1.43	0.21	ns
FLT	0.20	0.23	0.87	0.33	ns
G-CSF	0.27	0.09	3.00	0.02	ns
Granzyme B	0.05	0.04	1.25	0.29	ns
IFN-β	0.57	0.35	1.63	0.18	ns
IL-17E	0.59	0.32	1.84	0.09	ns
IL-9	0.02	0.03	0.67	0.27	ns
IL-3	0.06	0.05	1.20	0.36	ns
IL-33	0.04	0.03	1.33	0.25	ns
PDGF-AA	1.00	1.10	0.91	0.41	ns
PDGF-AB/BB	0.22	0.13	1.69	0.15	ns
TGF-α	0.21	0.04	5.25	0.01	0.03
TRAIL	0.09	0.04	2.25	0.07	ns
VEGF	2.39	0.04	59.75	<0.001	<0.001
FGF	0.005	0.03	0.17	0.004	0.01

The secretion index was calculated as the ratio of protein concentration in conditioned medium to that in corresponding cell lysates (medium/lysate) for each independent experiment. Values are presented as median index values derived from three independent biological experiments (n = 3) each performed in technical triplicate (three technical replicates per experiment). Fold change (FC) represents the ratio of index values in MDA-T32 relative to SCC147 cells. Statistical significance was assessed using the Mann–Whitney U test (two-tailed). To correct for multiple comparisons, false discovery rate (FDR) adjustment was applied using the Benjamini–Hochberg method. Differences were considered statistically significant at $p < 0.05$ and $q < 0.05$. "ns" indicates non-significant differences.
